# A Strategic Program for Risk Assessment and Intervention to Mitigate Environmental Stressor-Related Adverse Pregnancy Outcomes in the Indian Population

**DOI:** 10.3389/frph.2021.673118

**Published:** 2021-05-28

**Authors:** Divyanu Jain, Ajay K. Jain, Gerlinde A. S. Metz, Nina Ballanyi, Abha Sood, Rupert Linder, David M. Olson

**Affiliations:** ^1^Division of Reproductive Sciences, Department of Obstetrics and Gynecology, University of Alberta Faculty of Medicine and Dentistry, Edmonton, AB, Canada; ^2^Department of Obstetrics & Gynecology and In-vitro Fertilization Center, Jaipur Golden Hospital, New Delhi, India; ^3^IVF Center, Muzaffarnagar Medical College, Muzaffarnagar, India; ^4^Department of Neuroscience, University of Lethbridge, Lethbridge, AB, Canada; ^5^Specialist for Gynecology, Obstetrics, Psychosomatics and Psychotherapy, Birkenfeld, Germany; ^6^Departments of Pediatrics and Physiology, University of Alberta, Edmonton, AB, Canada

**Keywords:** environment, stress, pregnancy, adverse pregnancy outcomes, resilience, India

## Abstract

**The Problem:** Global environmental stressors of human health include, but are not limited to, conflict, migration, war, natural disasters, climate change, pollution, trauma, and pandemics. In combination with other factors, these stressors influence physical and mental as well as reproductive health. Maternal stress is a known factor for adverse pregnancy outcomes such as preterm birth (PTB); however, environmental stressors are less well-understood in this context and the problem is relatively under-researched. According to the WHO, major Indian cities including New Delhi are among the world's 20 most polluted cities. It is known that maternal exposure to environmental pollution increases the risk of premature births and other adverse pregnancy outcomes which is evident in this population.

**Response to the Problem:** Considering the seriousness of this problem, an international and interdisciplinary group of researchers, physicians, and organizations dedicated to the welfare of women at risk of adverse pregnancy outcomes launched an international program named Optimal Pregnancy Environment Risk Assessment (OPERA). The program aims to discover and disseminate inexpensive, accessible tools to diagnose women at risk for PTB and other adverse pregnancy outcomes due to risky environmental factors as early as possible and to promote effective interventions to mitigate these risks. OPERA has been supported by the Worldwide Universities Network, World Health Organization (WHO) and March of Dimes USA.

**Addressing the Problem:** This review article addresses the influence of environmental stressors on maternal-fetal health focusing on India as a model population and describes the role of OPERA in helping local practitioners by sharing with them the latest risk prediction and mitigation tools. The consequences of these environmental stressors can be partially mitigated by experience-based interventions that build resilience and break the cycle of inter- and-transgenerational transmission. The shared knowledge and experience from this collaboration are intended to guide and facilitate efforts at the local level in India and other LMIC to develop strategies appropriate for the jurisdiction for improving pregnancy outcomes in vulnerable populations.

## Introduction

Preterm birth (PTB) occurs before 37 weeks of gestation and is one of the leading causes of infant mortality in the world (1). The common causes for PTB include multiple pregnancies, intrauterine infection, diabetes, and preterm premature rupture of membranes (PPROM); however, most PTBs occur spontaneously (2). Defining the risk factors for PTB and other adverse pregnancy outcomes with population-specific categories is important for several reasons. First, it helps to identify women at risk in a particular region even before pregnancy; second, it can be useful to provide interventions before these women conceive; and third, subsequent pregnancies and neonatal outcomes in these populations can be improved by resilience-building strategies.

Human health is invariably affected by the changes in the environment that directly and indirectly influence the physical and mental well-being of every individual. Among the most vulnerable phases of human life to various environmental influences inducing stress in the mother and her developing fetus or newborn (3) are preconception, pregnancy, postpartum, and the neonatal period. Environment-related stressors may be classified as geographical (natural disasters, climate change, pollution, and pandemics) and political (migration, conflicts, and war). This review article discusses the role of environmental stressors in adverse pregnancy outcomes, particularly PTB, with a special focus on the Indian population and outlines the efforts of an international, interdisciplinary program aimed at mitigating the adverse effects of these stressors on maternal and newborn health. Each year ~27 million babies are born in India out of which 3.5 million are born preterm (4). The latest report on the Covid19 pandemic by the World Health Organization (WHO), United Nations Children's Fund (UNICEF), and the United Nations Population Fund (UNFPA) said that India is projected to record the greatest increase in numbers (more than 490,000) in maternal and child deaths in South Asia in spite of spending billions of dollars in testing and healthcare (5). However, it is unknown whether Covid19 pandemic will alter the total annual births or percentage of PTBs in the country. Therefore, the overall objective of this article is to encourage and bring together experts, policy makers, and funding bodies on this international strategic program that is of global value and will transform the current state of healthcare programs for women and children in low- and middle-income countries including India.

## Methodology

A literature search was performed with PubMed, Google Scholar and Google search using the keywords- “environment,” “stress,” “pregnancy outcomes,” “preterm birth,” “pollution,” and “India.” We included peer-reviewed research articles in the English language that were relevant to the topic. Additional references including book chapters and reports were retrieved manually from the cited references.

## The Opera Program

OPERA (Optimal Pregnancy Environmental Risk Assessment) is an international, interdisciplinary program that has received support from the Worldwide Universities Network (WUN), the WHO, and the March of Dimes (USA). The program comprises an international group of researchers, physicians and health care providers, foundations, and agencies dedicated to women at risk for preterm birth and other adverse pregnancy outcomes. The members of this program are committed to work with the local healthcare providers and disseminate knowledge to identify the women at risk using inexpensive and accessible techniques.

One of the aims of OPERA is to establish international collaborations between developed and developing countries to study the vulnerable populations affected by natural disasters, climate change, migration, pollution, and other environmental stressors including the recent coronavirus pandemic. OPERA organizes interactive conferences and workshops for health professionals working in local jurisdictions to help them learn about the latest advancements in pregnancy risk prediction models, share ideas and information on the hardships and obstacles in their respective jurisdictions related to pregnancy monitoring and care, and discuss strategies to identify those risk factors to improve the pregnancy outcomes. OPERA recently organized several such regional conferences in India. OPERA India is an ambitious initiative seeking to reduce the incidence of PTB and other adverse pregnancy outcomes in the country. This program plans to involve a transdisciplinary team including obstetricians, pediatrics, psychotherapists, nursing staff, policymakers, and epidemiologists to address the health challenges at the local level, share wisdom and work collaboratively to achieve OPERA's objectives.

The major aims of OPERA India:

1. To plan and develop collaborative studies in India with a special focus on the population in Delhi and the National Capital Region (NCR). They represent a culturally diverse population of immigrants from all parts of India with complex environmental conditions and lifestyles.
2. To identify from this population those with the highest risk for PTB based on environmental, cultural, social, familial, and economic backgrounds.
3. To develop risk prediction tools and strategies that are inexpensive and feasible to use for Indian healthcare professionals.
4. To develop simple and scalable interventions that meet the challenges of both the environment and the pandemic.

## Preterm Birth: An Indian Perspective

Preterm birth is a global problem, and it is an excellent example of an adverse pregnancy outcome. It is estimated that out of 12.9–15 million PTBs occurring each year; 60–85% of these occur in Asia and Africa (6, 7). Among all countries in 2010, India ranked highest with more than 3.5 million PTBs (7, 8). The Indian population is highly diverse in terms of cultures, socio-economic backgrounds, occupations, lifestyle, and environment. Emerging data show that these factors have been associated in part with the etiology of PTB (9). Both the alarming levels of environmental pollutants and the impact of the COVID-19 pandemic—especially on mental health—affect nearly everyone.

### Environmental Pollution and Pregnancy: Risks and Repercussions

In a community surrounded by rapid urbanization and industrialization, it is impossible to remain unexposed to high levels of chemicals and other pollutants (10). In 2019, an estimated 1.67 million deaths in India were attributable to air pollution that accounted for ~17.8% of the total annual deaths in the country (11). The consistently high level of pollution in India is a major public health concern and significantly impacts the life expectancy of this population (12). Environmental or occupational exposure to pollutants is a well-known co-factor in the etiology of several diseases and its impact on pregnancy outcomes is well-known. Numerous studies have indicated that exposure to potential toxicants in the air including cigarette smoke (13–15), contaminated drinking water (16), as well as occupational exposures (17) are associated with PTB. In a recent epidemiological study from three south Asian countries including India, it was found that between 2000 and 2016 an estimated 349,681 pregnancy losses per year were attributed to gestational exposure to ambient pollutants such as particulate matter (PM2·5) (18). In New Delhi, the capital of India and home for nearly 16 million people, the air quality has been consistently deteriorating due to vehicular pollution and exhausts from thermal power plants (19, 20). The persistent exposure to these toxins significantly affects male and female fertility (21), *in-utero* fetal development, and maternal health during pregnancy (15). Convincing evidence is available to prove that environmental pollutants and an unhealthy lifestyle can induce oxidative stress and generate reactive oxygen species (ROS) that adversely influence the *in-utero* fetal development and increases the susceptibility of the offspring to many diseases (22). India has also observed an increased demand for assisted reproductive techniques (ART) in the past few years (23), indicating a rise in cases of infertility among the reproductive age group who are constantly exposed to a variety of environmental stressors especially air pollution. While there are studies indicating that ART may increase the risk of PTB (24, 25), these complex and expensive procedures might also be one of the causes of prenatal maternal stress.

### Prenatal Maternal Stress

Besides environmental pollution, there are numerous other “modifiable” factors that can potentially contribute to PTB such as lifestyle, occupational stress, poverty, abuse, migration, and rapid urbanization; particularly affecting the populations residing in middle-income countries (26–28). According to the WHO, perinatal distress including mental stress, depression, or any form of anxiety related to pregnancy or the newborn is more common in lower-middle-income countries compared to high-income or developed countries (29). In India, maternal mental health services are either deficient or inaccessible in most regions of the country. In a meta-analysis, the prevalence of postpartum depression in India was reported 22% and the risk factors included financial instability, birth of a female child, low maternal education, and pregnancy complications (30). A study from South India reported that physical and psychological abuse, domestic violence, maternal depression, anxiety as well as low maternal education were all significantly associated with an increased risk of PTB (31). In Goa, a small province on the southwestern coast of India, maternal psychological morbidity was identified as an independent risk factor associated with low birth weight (<2.5 kg) (32). Exposure to adverse life events during pregnancy or early childhood is associated with the development of non-communicable diseases with disastrous consequences (33). Therefore, it is necessary to identify these “modifiable” risk factors at the clinical, individual, and population-level in low-resource settings (34).

### Endemic Diseases and Adverse Pregnancy Outcomes

In the Indian subcontinent, diseases like malaria are highly endemic in areas with prolonged warm and humid weather. A study from the southern Indian state of Karnataka involving 105 pregnant women revealed that babies born to women infected with malaria during pregnancy had significantly low birth weight compared to those born to healthy controls (35). The investigators also observed that 77.5% of the infected pregnant women were immigrants and more than 80% were economically poor and resided in rural areas. Dengue is another tropical disease found across India during the summer season. A hyperendemic area in the southern state of Kerala reported an increased risk of preterm births in women infected with dengue during pregnancy (36). The findings from these studies also indicate that women residing in rural areas are more susceptible to infections and associated pregnancy complications. It is well-known that infections before or during pregnancy are associated with a high risk of adverse pregnancy and neonatal outcomes; however, intermediate-risk factors such as poor socioeconomic status, poor sanitation, and low literacy may also contribute to the increased incidence of these infections.

### Population-Specific Challenges

The slow progress in reducing PTB rates can be partly attributed to the fact that there is a lack of resources for the healthcare professionals for early identification of women at risk of PTB which compromises the opportunities for early interventions. A recent study by Rai et al. emphasized the importance of social determinant factors, particularly maternal education and regular income in rural communities to mitigate the risk of PTB (37). The authors suggest that there is a need to implement effective public health programs for the uneducated and poor families residing in rural and remote regions of the country to improve pregnancy and newborn outcomes in underdeveloped areas. A low incidence of PTB (5.8%) has been observed in a small region of India where a majority of the women follow a healthy lifestyle, have access to regular antenatal check-ups, and deliveries are conducted in hospital settings (38). Over the years, risk prediction for PTB has relied upon the clinical assessment of the patient (39, 40); therefore, the best predictor to date is a previous history of PTB. There is an urgent need to combine early prediction tools with population-specific interventions to improve pregnancy outcomes at the local level (34).

## Experience-Based and Prospective Interventions

Maternal mental well-being and stress during pregnancy represent critical determinants of pregnancy outcomes and PTB risk. For example, maternal depression and psychological stress may trigger a pro-inflammatory state, doubling the risk of PTB and adverse birth outcomes (27, 41–43). Maternal depression and anxiety can also negatively impact mother-child bonding and child development in the long term (44–46). These observations are supported by experimental studies suggesting that chronic stress and cumulative lifetime stresses, prenatal, and intergenerational trauma can affect birth outcomes (27, 47). These associations emphasize the need to develop effective strategies that reduce stress to improve maternal mental health and ultimately promote both maternal and child health outcomes.

A range of evidence-based interventions has been adapted to the treatment of pregnant women at risk, showing promise in reducing maternal stress and mental strain to promote a healthy pregnancy, even reducing preterm delivery in high-risk mothers by about 50% (48, 49). For example, psychosomatic interventions such as psychotherapeutic and psychosocial interventions have been effectively used for the treatment of postpartum depression (50). The trend to integrate personalized psychotherapeutic and psychosocial treatments into clinical practice has provided evidence that stress reduction reduces the risk of pre-and postpartum depression with long-term benefits for maternal and child health (51–53). Even short-term interpersonal psychotherapy can promote maternal mental well-being and child health outcomes (53–55). Reducing maternal stress via psychotherapeutic approaches was shown to encourage positive birth outcomes (48, 56–59).

Integrated psychosomatic therapy is particularly effective in high-risk populations. A study in African-Americans showed that reducing behavioral and psychosocial risk factors improved pregnancy outcomes among high-risk women (60). A recent study in a vulnerable population in Pforzheim, Germany, showed that supplementary psychotherapy in addition to standard obstetrical care was able to reduce the risk of PTB by about 75% (61). While this therapy involved personalized and system-oriented psychosomatic therapy, solution-focused therapy, salutogenesis and couple therapy, it significantly improved pregnancy outcomes and newborn health, including birth weight (61). These findings indicate that psychosomatic interventions are important milestones in building maternal resilience and reduce the risk of adverse birth and newborn health outcomes.

## A Practical Intervention Model Based on the Experience of Die Pforzheim Studie, an Opera Project in Germany

### Early Life Experiences Influence Adulthood Personality

**Figure 1:**
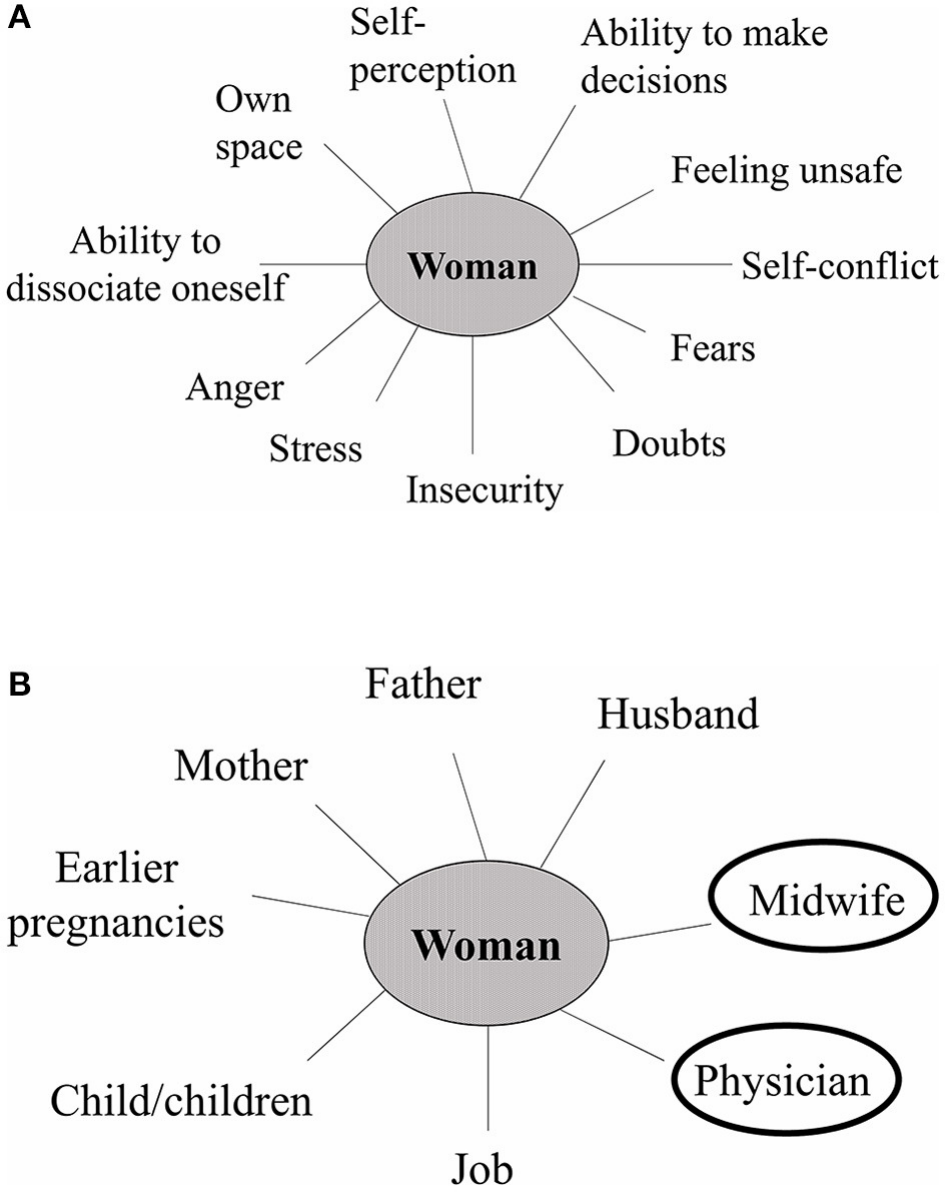
The psychological well-being of a woman during pregnancy depends on the combination of her internal emotions and external relationships. **(A)** Internal emotions. A pregnant woman experiences a wide range of internal emotions which are partly influenced by her own childhood experiences and partly by the physiological changes of pregnancy. **(B)** External relationships. Resilience and stress both can originate from a woman's social relationships. Social support from family, friends as well as healthcare providers is particularly important to promote maternal mental well-being.

Pregnancy is a phase of tremendous change in a woman's life as she braces herself for the rising tide of emotions and physiological changes in her body. A long clinical experience with pregnant women requiring psychotherapeutic support has shown that during pregnancy a woman emotionally goes through two parallel experiences: memories of her early childhood events and the present development of the child within her. For example, if a woman has experienced severe conflict with her own mother as a child, she relives those memories during her pregnancy on a very preconscious level (62, 63). In other words, she is surrounded by a range of internal emotions such as anger, fear, insecurity, and inability to make decisions (Figure 1A). These maternal childhood experiences influence a woman's perinatal mental health and even increase the risk of depression (64). On the other hand, a similar situation may occur when a woman comes together with her partner to form a family. In this union of two distinct individuals, two emotional systems merge because the woman's partner has also developed through his own emotional life experiences. Therefore, the emotional and stress experiences of previous generations can have a great impact on both of the parents (28, 65).

Frank Lake, a British psychiatrist and theologist, explained “Maternal-Fetal Distress Syndrome" and its association with fetal behavior from a psycho-socio-biological view (66). According to Lake, the “umbilical-effect” through the placenta or the emotional exchanges of physical and psychological feelings between the mother and her fetus during intrauterine life can contribute to personality disorders and might also affect subsequent pregnancies. From the viewpoint of prenatal psychology, prenatal development includes four stages: the Early Triad of conception, implantation, and discovery followed by the mother-baby bond in pregnancy (67). These stages are not well-known or scientifically researched, yet they are important, as they occur prior to the classical post-birth psychoanalytic development and involve intense feelings from the mother, baby, and the father, thereby forming basic patterns in the unconscious mind of an individual. Following birth, a child undergoes the well-known developmental psychological phases. A complex situation such as impending preterm birth can therefore be viewed as an entirety of physical and emotional processes.

### External Relationships-Stress or Support?

The type of social environment during pregnancy is particularly important as it can either positively or negatively impact the pregnancy outcome. Resilience and stress both can originate from a woman's external relationships (Figure 1B) as well as her internal emotions (Figure 1A). In the case of any adverse event during pregnancy, the earliest help may be found in the alertness of the care-takers or social support by the partner, family, and other social relationships. Young expectant parents with an active social and professional life might be more susceptible to various stressors in life (68). Research shows that social and familial support to young couples during pregnancy is associated with improved parenting efficacy (69).

Social assistance from the midwife and physician can be as important as familial support. It offers a deeper and timely understanding of complex situations relevant to the health of an individual. A practical intervention in the form of a multidisciplinary quality circle (QC) is practiced in the city of Pforzheim, Germany. The population was severely impacted during World War II due to destructive bombing and, with Germany's acceptance of Middle East migrants and others for the past 50 years, over 50% of its residents today are immigrants (70). The aim of QC is to involve an interdisciplinary group of professional health care providers including medical doctors, nurses, midwives, psychologists, social workers as well as other non-medical staff who collectively address the woman's social, medical, and psychological issues. This integrated approach enables provider group cooperation and understanding which is required for better pregnancy risk management and patient satisfaction.

An excellent example of the effectiveness of the QC is their approach to the Yazidi community and culture of about 3,600 individuals residing in Pforzheim. The Yazidis have been persecuted in Iraq for centuries as a religious minority, traumatized and burdened with extreme psychological distress (70). Given the long history of genocides against the Yazidis, personal recognition of a child before birth is uncommon, unlike western societies. Not until a newborn survives the crucial phases of fetal and newborn health risk is he/she recognized as a person or rather a Yazidi. This survival strategy among this small immigrant community is based on superficial adaptation and a very strong group identity (70). Therefore, it is necessary for care providers to be aware of the religious values and cultural practices of their local communities and appreciate how they affect the mindset of a pregnant woman.

## Using Psychotherapy and Social Interventions to Improve Pregnancy Outcomes in India

**Figure 2:**
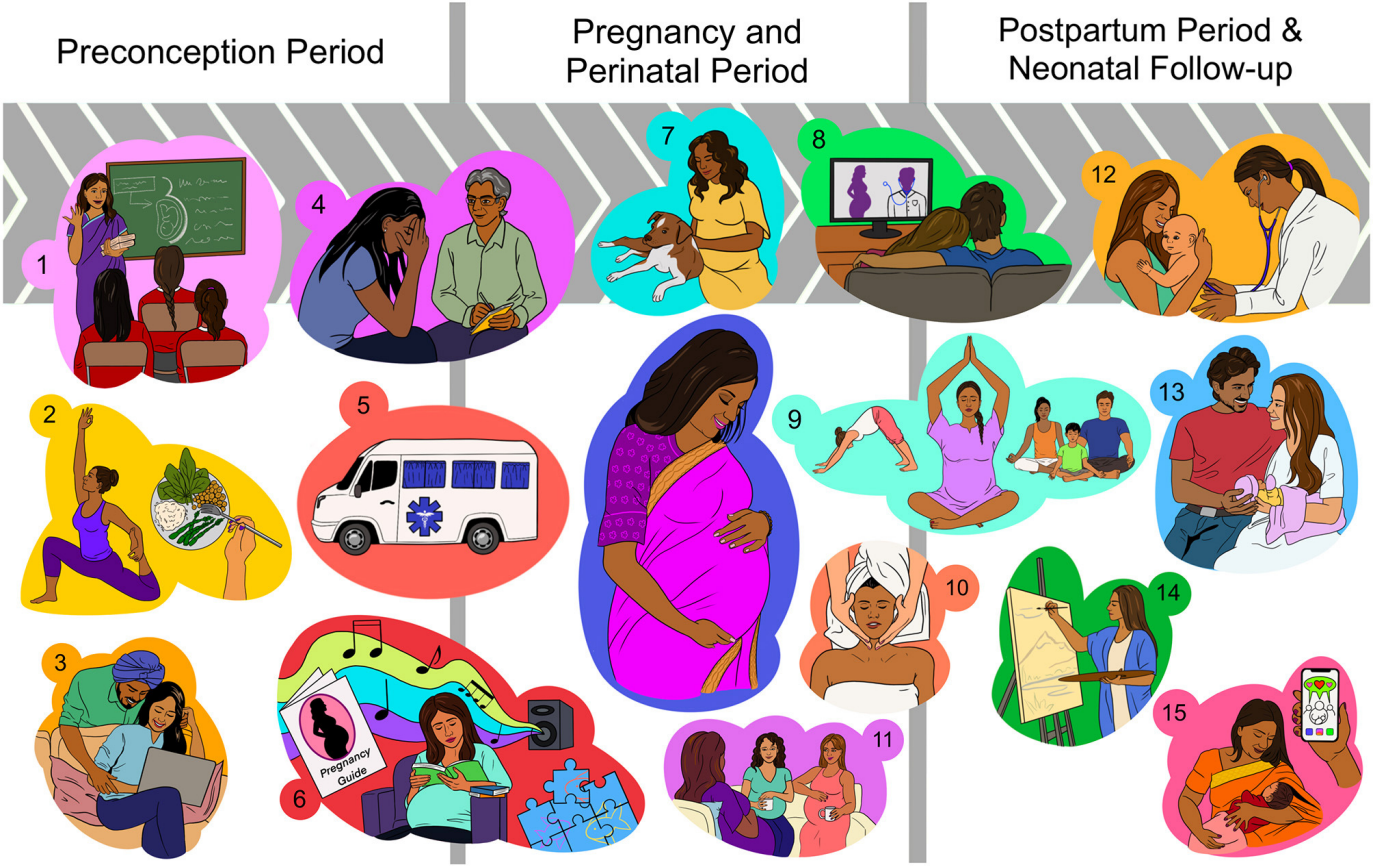
Proposed interventions to promote healthy pregnancy outcomes and reduce maternal stress before and during pregnancy, and after delivery. **(A)** Preconception period: (1) Providing health education in schools, colleges, and awareness programs to prepare women for their reproductive life; (2) Providing counseling on nutrition and lifestyle modification; (3) Educational webinars and counseling for young couples planning pregnancy; **(B)** Pregnancy and perinatal period: (4) Mental health counseling during each antenatal visit; (5) Mobile health clinics for public awareness, diagnosis, referrals, and emergency care in remote areas; (6) Practicing relaxing activities such as listening to music, reading a book, knitting, gardening, walking in a park, puzzle-solving, and setting goals. Providing free pregnancy handbooks (with easy-to-understand pictorial information) in public places such as supermarkets, shops, bus stops, and train stations; (7) Spending time with a pet animal; (8) Watching pregnancy health documentaries or a comedy show with the family to lighten up the mood; (9) Practicing pregnancy yoga, meditation, and moderate exercises; (10) Massage therapy for both the parents; (11) Socializing with other pregnant women and sharing each other's feelings; **(C)** Postpartum period: (12) Encouraging regular neonatal health check-ups; (13) Maternal and paternal stress counseling and guidance for mind exercises, relaxing activities to mitigate the stress; (14) Expressing inner thoughts through painting, expressive writing; (15) Joining nursing mothers' groups and other social support groups on social media and sharing each other's experiences.

Psychotherapeutic approaches and social interventions can be advantageous if they are introduced in the preconception period, continuing up to the postpartum and neonatal periods (Figure 2). In the preconception phase, the target population may include students attending schools and universities; as well as young couples planning to start a family. “Preconception” is a crucial period that can be rightly used to modify the risk factors and address pre-pregnancy health concerns/risks that could have negative maternal and fetal outcomes, especially in low-income countries (71). Provision of personal health education and generating awareness about pregnancy complications, healthy lifestyle, and mental health can make a positive difference and accelerate improvement in maternal-fetal health (72). In Japan, one of the countries with the highest standards of healthcare in the world, the provision of a pregnancy handbook to every pregnant woman since 1966 has been a simple, yet powerful tool of communication between the pregnant woman and healthcare provider (73, 74). Healthcare innovations such as mobile health clinics are useful and acceptable models for providing emergency and quality healthcare to pregnant women residing in rural and remote locations (75).

Psychotherapy and associated approaches include a significant social component. It was shown that maternal social environments determine fetal development and birth outcomes, especially amongst ethnic minorities (42, 76). As supported by experimental work (77, 78) and clinical studies (79, 80), fostering positive social interactions can significantly promote coping strategies and alleviate the impact of cumulative stress. Massage therapy may incorporate a social component that may be responsible for its effectiveness in improving birth outcomes (81). In addition, it represents a form of tactile stimulation, which was shown to dampen an activated stress response (82, 83), reduce symptoms of depression (81, 84), and modulate gene expression linked to immune functions (85, 86), thus potentially changing preterm birth risk. In terms of healthcare, over the years India has moved along an impressive path; however, the accessibility to these resources is limited in many underdeveloped regions due to the wide geographical distribution. Therefore, simple interventions during pregnancy such as massage therapy, social interaction among pregnancy groups, spending time with pet animals, and relaxing activities such as painting, puzzle-solving, and listening to music can be suggested to every pregnant woman during the antenatal visits which might at least partly help in coping with the emotional and mental stress associated with pregnancy (Figure 2).

A different approach to reduce stress involves targeted relaxation techniques, such as progressive muscle relaxation, yoga, and mindfulness to improve stress coping in pregnant women (56, 81, 87, 88) and support fetal development (89). Mindfulness-based interventions can reduce stress during pregnancy thus reducing the risk of birth complications, such as PTB and cesarean section (44, 88, 90, 91). Moreover, mindfulness and gratitude-based interventions were suggested to weaken prenatal stress and promote psychological and physical maternal well-being (57). An advantage of these therapies is their potential to use the internet and web-based tools (88, 92), which enables cost-effective and personalized applications also in remote communities and during the ongoing COVID-19 pandemic. In a virtual world, social support groups on popular social media platforms can be a cost-effective way for reaching out to women before, during, and post-pregnancy to address their concerns and help them connect with women facing similar situations. Similar considerations apply to other non-pharmacologic therapies such as cognitive behavioral therapy (93, 94) or expressive writing (94–96).

Clinical studies support the notion that psychosomatic or behavioral interventions during pregnancy are potent means to reduce maternal perceived stress and promote maternal mental health positive birth outcomes (49, 56, 57, 97). However, with the complex global health challenges such as the recent novel coronavirus pandemic; prevention of adverse pregnancy outcomes requires a multi-dimensional approach. As such, OPERA's research objectives will use an inter-disciplinary focus employing basic and clinical research to develop risk prediction tools and resilience-building strategies relevant to the needs of the target population.

## Conclusion

The evidence is sufficient to determine a causal relationship between environmental stressors such as pollution, pandemics, climate change, migration, and adverse pregnancy outcomes including preterm birth. Increasing awareness and strategically planning to provide timely interventions to the vulnerable populations is the need of the hour especially in low-resource settings. OPERA will strengthen the efforts in India to develop international collaborations and enhance efforts at the local level to achieve the positive effects of the proposed interventions for improving pregnancy outcomes and reducing PTBs in this region.

## Author Contributions

DJ contributed to the conception and design of the review, and writing of the manuscript. AJ, GM, and RL contributed to the writing and critical revision of the manuscript. NB contributed to the figure designing and format analysis. AS provided guidance throughout the writing of the manuscript. DO contributed to the design of the review, funding acquisition, writing, and critical revision of the intellectual content of the manuscript. All authors approved the final manuscript.

## Conflict of Interest

The authors declare that the research was conducted in the absence of any commercial or financial relationships that could be construed as a potential conflict of interest.
